# Mapping PSA density to outcome of MRI-based active surveillance for prostate cancer through joint longitudinal-survival models

**DOI:** 10.1038/s41391-021-00373-w

**Published:** 2021-05-06

**Authors:** Vasilis Stavrinides, Georgios Papageorgiou, Dominic Danks, Francesco Giganti, Nora Pashayan, Bruce Trock, Alex Freeman, Yipeng Hu, Hayley Whitaker, Clare Allen, Alex Kirkham, Shonit Punwani, Geoffrey Sonn, Dean Barratt, Mark Emberton, Caroline M. Moore

**Affiliations:** 1 UCL Division of Surgery & Interventional Science, University College London, London, UK; 2 The Alan Turing Institute, London, UK; 3 Department of Urology, University College London Hospitals NHS Foundation Trust, London, UK; 4 Department of Economics, Mathematics and Statistics, Birkbeck College, University of London, London, UK; 5 Institute of Cancer and Genomic Sciences, University of Birmingham, Birmingham, UK; 6 Department of Radiology, University College London Hospitals NHS Foundation Trust, London, UK; 7 Department of Applied Health Research, Institute of Epidemiology & Health, University College London, London, UK; 8 Division of Epidemiology, James Buchanan Brady Urological Institute, Johns Hopkins University, Baltimore, MD USA; 9 Department of Pathology, University College London Hospitals NHS Foundation Trust, London, UK; 10 Centre for Medical Image Computing, University College London, London, UK; 11 Wellcome EPSRC Centre for Interventional & Surgical Science (WEISS), University College London, London, UK; 12 Department of Medical Physics and & Biomedical Engineering, University College London, London, UK; 13 Centre for Medical Imaging, University College London, London, UK; 14 Department of Urology, School of Medicine, Stanford School of Medicine, Stanford, CA USA

**Keywords:** Prostate cancer, Outcomes research

## The need for dynamic risk prediction in MRI-based AS

The use of multiparametric magnetic resonance imaging (mpMRI) for the active surveillance (AS) of localised prostate cancers is increasing, and evidence suggests that mpMRI facilitates the selection of AS candidates while minimising the need for follow-up biopsies [1]. As the natural history of prostate cancer is not entirely defined, it is unsurprising that that many AS schedules remain prescriptive [2]. Regular, protocol-based biopsies condition participants on sampling scheme, allowing less biased inferences regarding the relationship between risk factors and disease progression in ways reminiscent of clinical trial design.

However, although this more rigid approach is reassuring to clinicians, it is antithetical to the principles of personalised medicine, where decisions on follow up or treatment should be dynamically informed by the unique longitudinal trajectory of each patient. In MRI-based AS this conflict can be demonstrated for prostate-specific antigen (PSA) or PSA density (PSAD): although both have been associated with progression or treatment, existing studies predominantly focus on baseline PSA or PSAD values rather than longitudinal trends, which are more clinically relevant over surveillance periods that often span several years. In part, this shortfall can be attributed to methodological limitations; standard logistic regression is not ideal for dealing with longitudinal measurements, whereas extended Cox models assume piecewise-constant, measurement error-free trajectories for time-varying covariates and are not optimal for modelling endogenous biomarkers such as PSAD [3–5].

Dynamic risk prediction methods could address this need. A good example is joint longitudinal-survival models: these have a distinct advantage over traditional survival analyses, as they consider all longitudinal measurements of a predictor (e.g. PSAD) and account for variability at the level of the individual when predicting an event, which could be very useful in AS settings. 1To prove this concept, we visualised MRI-calculated PSAD trends and examined their association with event-free survival (EFS) under a joint longitudinal-survival analysis framework in a recently described AS cohort where regular biopsies were omitted in favour of MRI-led monitoring [6].

## Joint modelling of PSAD and outcome in an imaging-based AS cohort

The University College London Hospital AS cohort (*n* = 672) includes men with a baseline mpMRI, Gleason 3 + 3 or 3 + 4 prostate cancer, and PSA < 20 ng/mL. The monitoring protocol has been described elsewhere [6]. In brief, all men have mpMRI at baseline and 12 months; those with MRI-visible disease (i.e. Likert 4–5 or well-defined lesion) undergo an additional scan at 24 months. Beyond these time points, mpMRI is performed in cases of clinical suspicion or unexplained PSA fluctuations. PSAD values were obtained at baseline and with each follow-up mpMRI (up-to-date PSA divided by the prostate volume, estimated by the ellipsoid formula on MRI; Supplementary Table 1). The primary outcome was EFS, with “event” defined as any prostate cancer treatment, upgrading to Gleason ≥4 + 3 on follow-up biopsy, transition to watchful waiting or death. We used a linear mixed-effects longitudinal model with random intercepts for individuals and a random non-linear time effect using natural cubic splines to describe log_2_PSAD over time (months after baseline mpMRI). This was integrated into a Cox regression component with baseline Gleason and MRI visibility as predictors, in order to construct a joint model. Inference was carried out using both Bayesian and maximum likelihood-based estimation approaches [3, 4]. All analyses were performed in R (R Foundation for Statistical Computing, https://www.R-project.org/) and *p* values, where obtained, were considered significant at the 0.05 level.

**Fig. 1:**
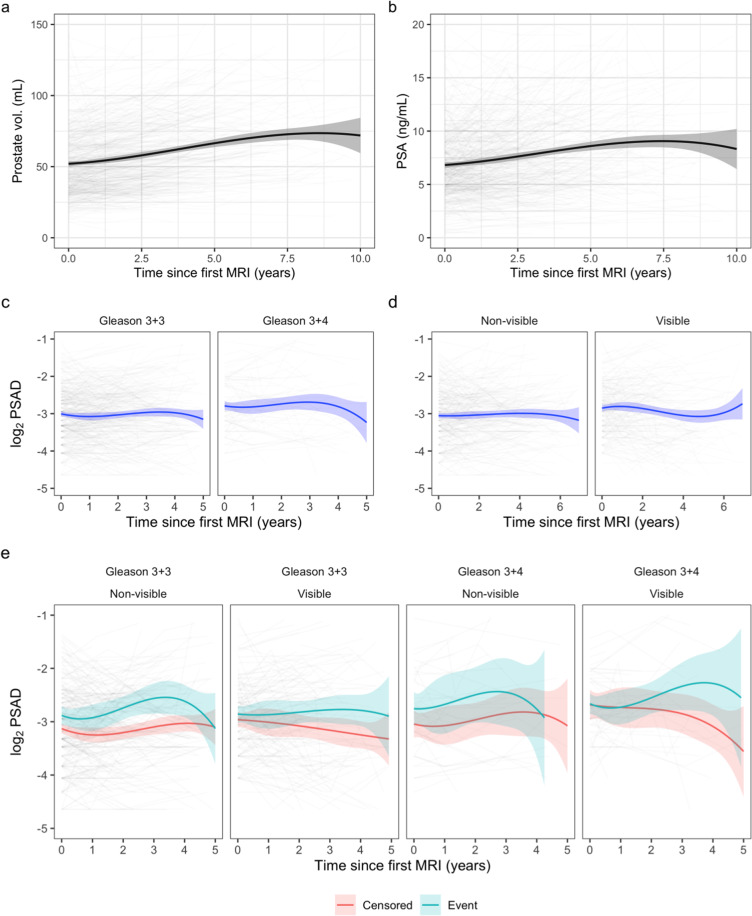
MRI-calculated prostate volume and PSAD trends over time. **a** Prostate volume. Volumes were calculated using the ellipsoid formula. There was a steady increase in mean prostate volume over time (spline curve shown), at an approximate average rate of 3.3 mL/year. **b** PSA. There was a steady increase in mean PSA (spline curve shown). **c**, **d** PSAD trends stratified by Gleason and MRI visibility. PSAD was higher at baseline and throughout surveillance in men with Gleason 3 + 4 and MRI-visible disease. **e** PSAD trends stratified by censoring status. PSAD in men who ultimately experienced an event was consistently elevated throughout AS compared to men who were censored. The downward trend in the latter years is likely due to the drop-out of men with higher PSAD who experience events as time progresses.

As previously reported, median follow up for the cohort was 58 months (IQR: 37–82), whereas for censored men median follow up was 63 months (IQR: 44–88). In total, 250 events were recorded, the vast majority being treatments or upgrading to Gleason ≥4 + 3 on follow-up biopsies. As expected, prostate volume and PSA increased over time (Fig. 1a, b). Men with Gleason 3 + 4 and higher PSAD at baseline had shorter EFS compared to men with 3 + 3 and lower PSAD, regardless of disease visibility on MRI (*p* < 0.01, log-rank test; Supplementary Fig. 1). Within Gleason groups, there were no EFS differences attributable to baseline PSAD (pairwise log-rank comparisons).

Interestingly, there was a non-linear PSAD trend over time, with PSAD being consistently higher throughout AS in men with Gleason 3 + 4 or visible disease (Fig. 1c, d) and men who ultimately experienced an event (Fig. 1e). Baseline Gleason grade and MRI visibility were significant predictors of EFS in a joint survival-longitudinal model incorporating the log_2_PSAD longitudinal component, with hazard ratios (HR) of 2.32 (95% CI: 1.75–3.08) and 1.93 (95% CI: 1.49–2.50) for Gleason 3 + 4/MR-visible disease, respectively. The HR for the log_2_PSAD association parameter (i.e. the Cox coefficient associated with longitudinal log_2_PSAD) was 1.77 (95% CI: 1.48–2.11), suggesting that each PSAD doubling is associated with a 1.77× risk increase. The value of this point estimate was invariant to the inferential method used (likelihood or Bayesian).

## Concluding remarks

Our findings formally describe the association of PSAD with clinical outcome in imaging-led AS and support the use of dynamic risk estimation for individualising the timing of follow-up tests or radical treatment. In addition, our results corroborate those of other authors who report higher rates of upgrading in men with MRI-visible Gleason 3 + 3 disease and high PSAD at baseline [7, 8]. The limitations inherent to our cohort have been described, principally its retrospective, single-centre nature and the avoidance of scheduled biopsies in favour of a personalised, risk-adjusted biopsy approach. Also, strictly speaking, transition to watchful waiting and non-prostate cancer-related death occurring before other events should not necessarily be considered as failure but as “AS graduation”. However, the overwhelming majority of events were treatment or upgrading-related (86.4%), and competing risk approaches were beyond the scope of this work. For the time being, we note that joint longitudinal-survival modelling can feasibly offer a dynamic risk estimation framework that should be considered in AS settings, where follow up is long and risk is constantly updated by new clinical, laboratory or imaging information. We observe that PSAD change prompts further assessments through imaging or biopsy and is, therefore, associated with outcome. Future research should expand on these considerations and explore the potential of multivariable models with additional longitudinal predictors beyond PSAD [9, 10].

## Supplementary information

Supplementary Figure and Table

## Footnotes

1 The traditional Cox regression equation is *h*_*i*_ (*t*) = *h*_0_ (*t*) exp{*γ*^*T*^ *wi*}, where *h*_0_(t) is the baseline hazard and *γ*^*T*^ *w*_*i*_ is a linear combination of predictors and their coefficients. By comparison, a joint model in its simplest form is expressed by *h*_*i*_ (t│*M*_*i*_(*t*)) = *h*_0_(*t*) exp{*γ*^T^ *w*_*i*_ + ***αm***_***i***_**(*****t*****)**}; the additional terms are ***m***_***i***_**(*****t*****)***,* the longitudinal history of the endogenous biomarker, and **α**, the association parameter expressing the strength of the relationship between ***m***_***i***_**(*****t*****)** and the event of interest (e.g. exit from AS).
