# Supplementary material for: Mapping PSA density to outcome of MRI-based active surveillance for prostate cancer through joint longitudinal-survival models
Source: Prostate Cancer Prostatic Dis. 2021 May 6;24(4):1028–31. doi: 10.1038/s41391-021-00373-w (PMC8616763; doi:10.1038/s41391-021-00373-w)
Supplement: Supplementary file 1 — Supplementary Figure and Table [file 41391_2021_373_MOESM1_ESM.pdf]

a

Cohort baseline characteristics (n=672)

Gleason	3+3		3+4	
n	524		148	
MRI-visible n (%)	212	(40.5%)	83	(56.1%)
Age (years)	62	(56-66)	64	(59-70)
pPSA (ng/mL)	6	(4.5-8.4)	6.9	(5.2-8.9)
PSAD (ng/mL^2)	0.12	(0.09-0.18)	0.14	(0.1-0.22)

b

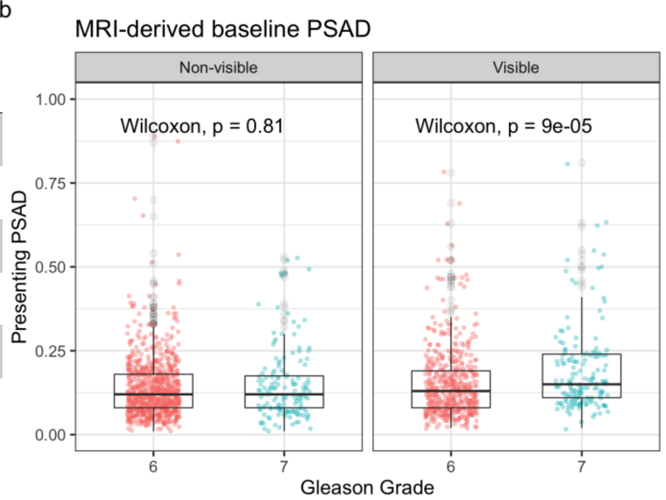

c

d

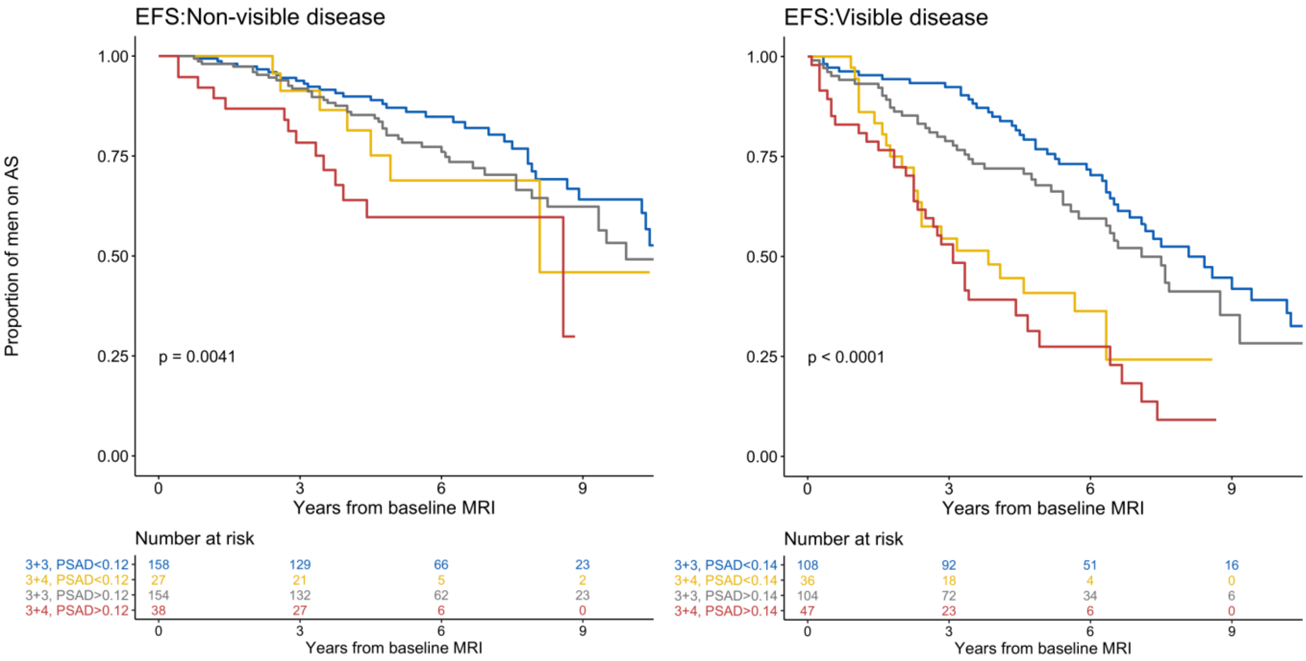

Supplementary Figure: Baseline Gleason, MRI-visibility and PSAD and clinical outcome. (a) Baseline characteristics for the cohort (n=672). More men with Gleason 3+4 cancer had visible disease at baseline compared to those with 3+3. **(a) PSAD at baseline.** PSAD differences according to MRI visibility were present within the Gleason 3+4 subgroup **(b,c) EFS stratified by Gleason, MRI visibility and PSAD (below and above group median).** EFS differences were present between different Gleason groups.

Supplementary Table: Number of MRIs (and PSAD measurements) by baseline Gleason and MRI visibility								
**Gleason**	**MRI-visible disease**		**Baseline**	**2**	**3**	**4**	**5**	**6**
**3+3**	**No**	**Months (IQR)**	-	14 (11-22)	34(25-45)	53 (41-65)	69 (55-94)	86 (72-106)
		**N**	294	280	235	185	104	41
	**Yes**	**Months (IQR)**	-	15 (11-24)	33 (24-43)	50 (38-61)	62 (51-78)	71 (61-86)
		**N**	193	187	150	117	69	37
**3+4**	**No**	**Months (IQR)**	-	13 (11-16)	28 (24-34)	40 (36-48)	51 (46-62)	65 (62-68)
		**N**	62	60	46	31	19	4
	**Yes**	**Months (IQR)**	-	12 (9-17)	25 (23-30)	39 (35-48)	55 (46-68)	74 (68-86)
		**N**	72	64	47	32	16	6

Supplementary Table: Number of MRIs performed for different Gleason and MRI visibility subgroups. A PSAD measurement was calculated after each MRI using the latest pre-imaging PSA value and the MRI-calculated prostate volume. Most patients underwent multiple measurements.
